# The Evolution of Randomized Clinical Trial Designs to Assess Therapeutics in Alzheimer Disease

**DOI:** 10.1001/jamanetworkopen.2025.29665

**Published:** 2025-08-29

**Authors:** Etienne Aumont, Joseph Therriault, Angela T. H. Kwan, Giovanna Carello-Collar, Saman Arfaie, Brandon J. Hall, João-Pedro Ferrari-Souza, Marcel S. Woo, Arthur C. Macedo, Cécile Tissot, Lydia Trudel, Nesrine Rahmouni, Stijn Servaes, Paolo Vitali, Jean-Paul Soucy, Tharick A. Pascoal, Eduardo R. Zimmer, Serge Gauthier, Pedro Rosa-Neto

**Affiliations:** 1 McGill University Research Centre for Studies in Aging, McGill University, Montréal, Quebec, Canada; 2 Department of Neurology and Neurosurgery, Faculty of Medicine, McGill University, Montréal, Quebec, Canada; 3 Faculty of Medicine, University of Ottawa, Ottawa, Ontario, Canada; 4 Graduate Program in Biological Sciences: Biochemistry, Universidade Federal do Rio Grande do Sul, Porto Alegre, Rio Grande do Sul, Brazil; 5 Department of Psychiatry, University of Pittsburgh School of Medicine, Pittsburgh, Pennsylvania; 6 Department of Neurology, University Medical Center Hamburg-Eppendorf, Hamburg, Germany; 7 Lawrence Berkeley National Laboratory, University of California, Berkeley; 8 Peter O’Donnell Jr Brain Institute, UT Southwestern Medical Center, Dallas, Texas

## Abstract

**Importance:**

The success of recent randomized clinical trials (RCTs) for Alzheimer disease (AD), particularly those focusing on anti-amyloid therapies, has been discussed at length. However, the evolution of RCT design features for AD that preceded this success remain underexplored.

**Objective:**

To describe temporal changes in the features of RCT design for interventions in AD.

**Evidence Review:**

PubMed, Scopus, and Web of Science databases were searched in January 2025 for phase 2 and 3 AD RCTs published between January 1992 and December 2024. RCTs that investigated an intervention for AD, with a placebo or standard-of-care control group, were included. Four assessors independently reviewed full-text articles to capture study characteristics.

**Main Outcomes and Measures:**

The number of participants and the duration of RCTs as well as the target population, outcomes, and funding were extracted from published reports. These features were analyzed with respect to time using linear regression and χ^2^ analyses.

**Results:**

The study included 203 RCTs with 79 589 participants testing interventions in AD. From 1992 to 2024, the mean sample size increased by 464% for phase 2 RCTs (from 42 to 237), and 50% for phase 3 RCTs (from 632 to 951), while the mean trial duration increased by 188% (from 16 to 46 weeks) for phase 2, and 256% (from 20 to 71 weeks) for phase 3 RCTs. This longer duration of RCTs may be partially attributed by a greater share of disease-modifying rather than symptomatic treatments. Similarly, more recent trials required AD biomarker evidence for enrollment (from 1 of 36 [2.7%] before 2006 to 40 of 76 [52.6%] since 2019). A substantial difference in the type of therapeutics researched was observed, with anti-amyloid and anti-tau RCTs being more likely to be funded by the pharmaceutical industry compared with neurotransmitter or other RCTs (anti-amyloid or anti-tau, 68 of 71 [95.8%]; neurotransmitter, 52 of 69 [77.6%]; other, 33 of 52 [63.5%]). RCT transparency improved, with more frequent data accessibility statements, registered reports, and better reporting on race and ethnicity.

**Conclusions and Relevance:**

This methodology research of AD RCTs highlights substantial changes in key features of AD clinical trials from 1992 to 2024. AD RCTs have become larger and longer, such that they are powered to detect smaller clinical differences. The increased sample sizes and duration should enable the detection of smaller and more slowly occurring outcomes, which may lead to successful RCTs of therapies with slower and more subtle efficacy.

## Key Points

**Question:**

What are the major characteristics of randomized clinical trials (RCTs) for Alzheimer disease (AD), and how have these characteristics evolved?

**Findings:**

In this study of 203 RCTs with 79 589 participants, the mean sample size and trial duration increased over time, reflecting a shift from symptomatic treatments toward disease-modifying therapies earlier in the disease. Disease-modifying RCTs tended to be funded by the pharmaceutical industry.

**Meaning:**

The findings of this study suggest that AD RCTs are now designed to detect smaller clinical differences, which emphasizes the need to define systematic tools to assess these interventions’ clinical meaningfulness.

## Teaser

This study describes temporal changes in how randomized clinical trials for new Alzheimer disease characteristics were designed from 1992 to 2024.

## Introduction

Randomized clinical trials (RCTs) are the gold standard to establish efficacy for new medical interventions against disease to improve patient care.^1^ Few age-related neurological disorders are as devastating as Alzheimer disease (AD). Despite hundreds of AD RCTs having been completed since the 1990s, only a select few have led to therapy approval.^2,3^ As the molecular bases of AD have been increasingly well understood, RCTs have aimed at disease-modification, which could shift the typical characteristics of AD RCTs.^4,5^ Characteristics such as sample size, study duration, outcome measure (biomarker and clinical), trial design, type of intervention, target population, and the funding source are also shaped based on RCTs’ aims, the funds, and the expected therapeutic effects. The evolution of pharmaceutical RCTs’ characteristics over time has been studied in other areas of medicine, identifying changes in study funding (from government agencies to the pharmaceutical industry),^6^ targeting earlier stages of the disease, and a shift toward biomarker end points to complement traditional clinical ones.^6,7^ Similar shifts may have occurred in AD research, but to our knowledge, no study has yet described temporal trends for AD RCT characteristics.

This study aims to describe changes in key design and methodological features of AD RCTs over the last 33 years. Our objectives are to describe temporal trends in (1) trial methods and reporting, including race and ethnicity; (2) primary end points selected; (3) the use of AD biomarkers as enrollment criteria and measures of target engagement; and (4) the proportion of studies funded by industry vs not-for-profit organizations. Such changes need to be understood and described to help clinicians have a better understanding of expected therapeutic effects and interpretations of clinical trials depending on when they took place.

## Methods

### Search Strategy and Study Selection

Using the methods of a meta-analysis to study the methods used within a field of research, we conducted this methodology research of phase 2 and 3 AD RCTs identified through a systematic literature search. Appropriate reporting guidelines for this type of study were followed.^8^ Exclusion and inclusion criteria were developed using the patient, intervention, comparator, outcome, and time frame (PICOT) framework, modeled on previous work in oncology (Table).^6,7,9,10,11,12^ A comprehensive search was performed using PubMed, Scopus, and Web of Science databases in January 2025 using specific search and selection terms determined based on the PICOT. Four authors (J.T., E.A., S.A., and A.T.H.K.) independently reviewed article abstracts for inclusion, with any disagreement resolved by consensus. In a second round, 4 assessors (J.T., E.A., A.T.H.K., and M.S.W.) evaluated full texts for inclusion and data abstraction.

**Table.** PICOT Table for RCT Selection

Element	Inclusion	Exclusion
Patient	AD and its continuum (including mild cognitive impairment and dementia of Alzheimer type)	Outside of the AD continuum, animal studies, in vitro or in silico studies
Intervention	Phase 2 or 3 clinical trial or corresponding to current definition of such trial; includes pharmacological and nonpharmacological interventions	Phase 1 trials, open-label extensions, unblinded trials
Comparator	Randomized placebo intervention (including placebo group or crossover design) or standard-of-care	Exclusively subgroup comparisons
Outcome	Main outcome of the trial reported; quantitative or semiquantitative outcome	Secondary analysis of RCT data, interim analyses, or qualitative outcome
Time frame	Publication date between 1992 and 2024	Outside of the targeted period

Abbreviations: AD, Alzheimer disease; PICOT, patient, intervention, comparator, outcome, and time frame; RCT, randomized clinical trial.

### Data Abstraction

We used a modified version of a previously published data abstraction tool to gather key RCT design and reporting practices.^9^ This included the study phase, number of randomized participants, study duration, primary outcomes, and inclusion criteria (ie, Mini-Mental State Examination [MMSE] and biomarker). Study sponsorship information was extracted based on explicit statements, following definitions proposed by previous work, into (1) funded by for-profit companies and (2) financed exclusively by not-for-profit organizations (including governmental agencies).^13,14,15^ Mechanism of action was first categorized as disease-modifying or symptomatic based on the consensus of 2 assessors (J.T. and P.R.-N.) according to the definitions provided by Cummings et al.^16^ Briefly, disease-modifying trials targeted key biological features of AD or were otherwise hypothesized to modify the trajectory of AD progression.^16^ Symptomatic trials included trials hypothesized to modify AD symptoms (cognitive or neuropsychiatric) without altering the underlying disease progression.^16^ A more precise intended mechanism of action (eg, cholinergic, amyloid-β) was also noted. When a primary or secondary outcome was intended to verify the mechanism of action, it was considered target engagement. If a trial included an open-label extension phase, only the randomized portion of the study was considered in the study duration. If an article reported more than 1 RCT, each trial was treated separately.

### Statistical Analysis

Studies were grouped into 3-year intervals based on the publication year to maximize the stability of trends while maximizing the granularity. Due to the limited number of published studies between 1992 and 2009, these windows were grouped in cases where the study number were too low, ie, 10 for fewer for frequency analyses (eFigure, A in Supplement 1). Temporal trends in RCT design, choice of outcome measures and reporting practices were evaluated using Welch-corrected *t* tests and Spearman correlations for continuous variables, and χ^2^ tests for categorical variables, with *P* < .05 considered statistically significant. Prism version 10.3.1 (GraphPad) was used to conduct analyses. The full dataset is available on Open Science Framework^17^ and in the eTable in Supplement 2.

## Results

### Duration and Sample Size

**Figure 1.**
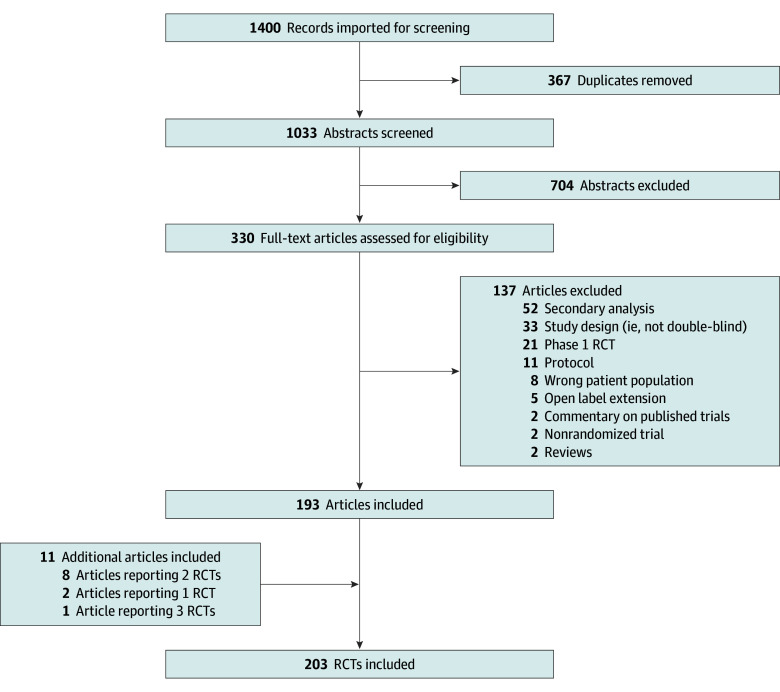
Study Selection Flowchart RCT indicates randomized clinical trials.

**Figure 2.**
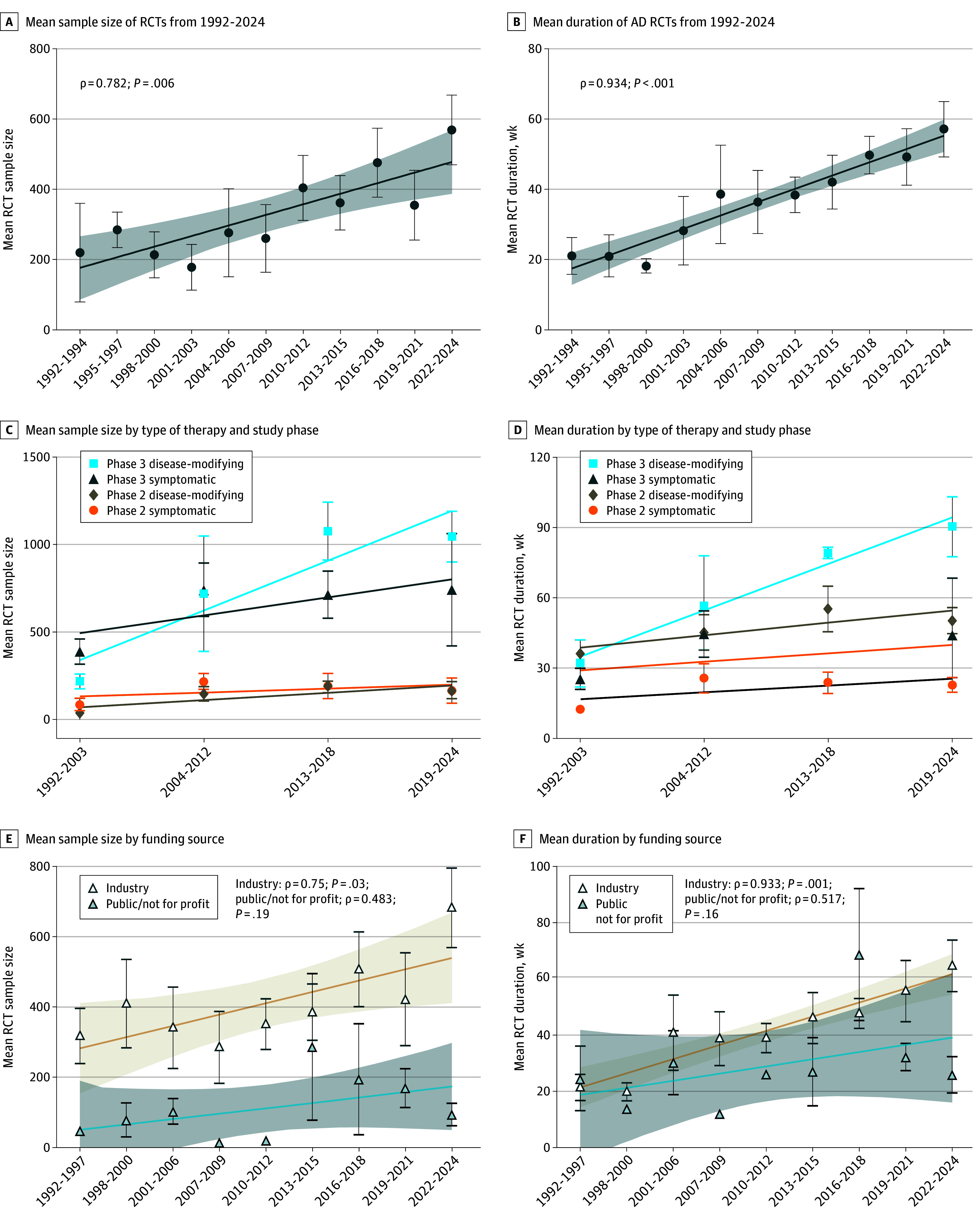
Trends in Randomized Clinical Trial (RCT) Sample Size Duration A, B, E, and F, Error bars show SE to the mean, while the shaded areas correspond to the 95% CIs of regression lines. C and D, Early time periods were grouped due to the low number of disease-modifying trials before 2007; error bars show SEs. AD indicates Alzheimer disease.

Among 203 RCTs including 79 589 individuals (Figure 1), we observed an increased sample size over the study period, from a mean (SD) of 220 (243) individuals in 1992 to 1994 to 569 (527) in 2022 to 2024 (Figure 2A). Similarly, the duration of RCTs increased from a mean (SD) of 21.0 (9.1) weeks in 1992 to 1994 to 58.2 (47.2) weeks in 2022 to 2024 (Figure 2B). Phase 3 trials were significantly longer and had larger sample sizes than phase 2 trials (duration: 59.8 vs 36.7 weeks; t_99.6_ = 3.559; *P* < .001; sample size: 818 vs 170 individuals; t_73.7_ = 8.302; *P* < .001). By separating them into subgroups, increases were observed for both phase 2 (sample size: from 42 to 237 individuals [464% increase]; ρ = 0.800; *P* = .005; duration: from 16 to 46 weeks [188% increase]; ρ = 0.864; *P* = .001) and phase 3 trials (sample size: from 632 to 951 individuals [50% increase]; ρ = 0.809; *P* = .004; duration: from 20 to 71 weeks [256% increase]; ρ = 0.918; *P* < .001). Therefore, the increased sample size and trial duration are generalized over phase 2 and phase 3 trials.

We hypothesized that the increasing proportion of disease-modifying trials would lead to longer trial durations. First, we found that disease-modifying trials have indeed been more frequent, particularly starting from the 2007 to 2009 period (eFigure, B in the Supplement). Disease-modifying trials were scarce between 1992 and 2006 (4 of 36 RCTs [11.1%]). After a rapid shift, more than half of the studies from 2007 to 2024 (95 of 168 RCTs [56.5%]) were disease-modifying trials. Second, disease-modifying trials were significantly longer than symptomatic trials (mean [SD] duration, 61.9 [39.4] weeks vs 26.1 [29.3] weeks; difference, 35.8 weeks; 95% CI, 26.1-45.6 weeks; t_178.6_ = 7.260; η^2^ = 0.228; *P* < .001). This finding applied to both phase 2 and phase 3 trials (Figure 2C and D). Third, we found that only industry-funded trials had significantly increased duration and sample size over time (Figure 2E and F). Industry-funded RCTs were also significantly longer and had larger sample sizes than not-for-profit (mean [SD] duration: 48.7 [41.9] weeks vs 30.3 [23.0] weeks; t_104.5_ = 3.647; *P* < .001; mean [SD] sample size: 467 [538] vs 152 [261 ]individuals; t_126.4_ = 5.175; *P* < .001).

### RCT Targets and Measures

**Figure 3.**
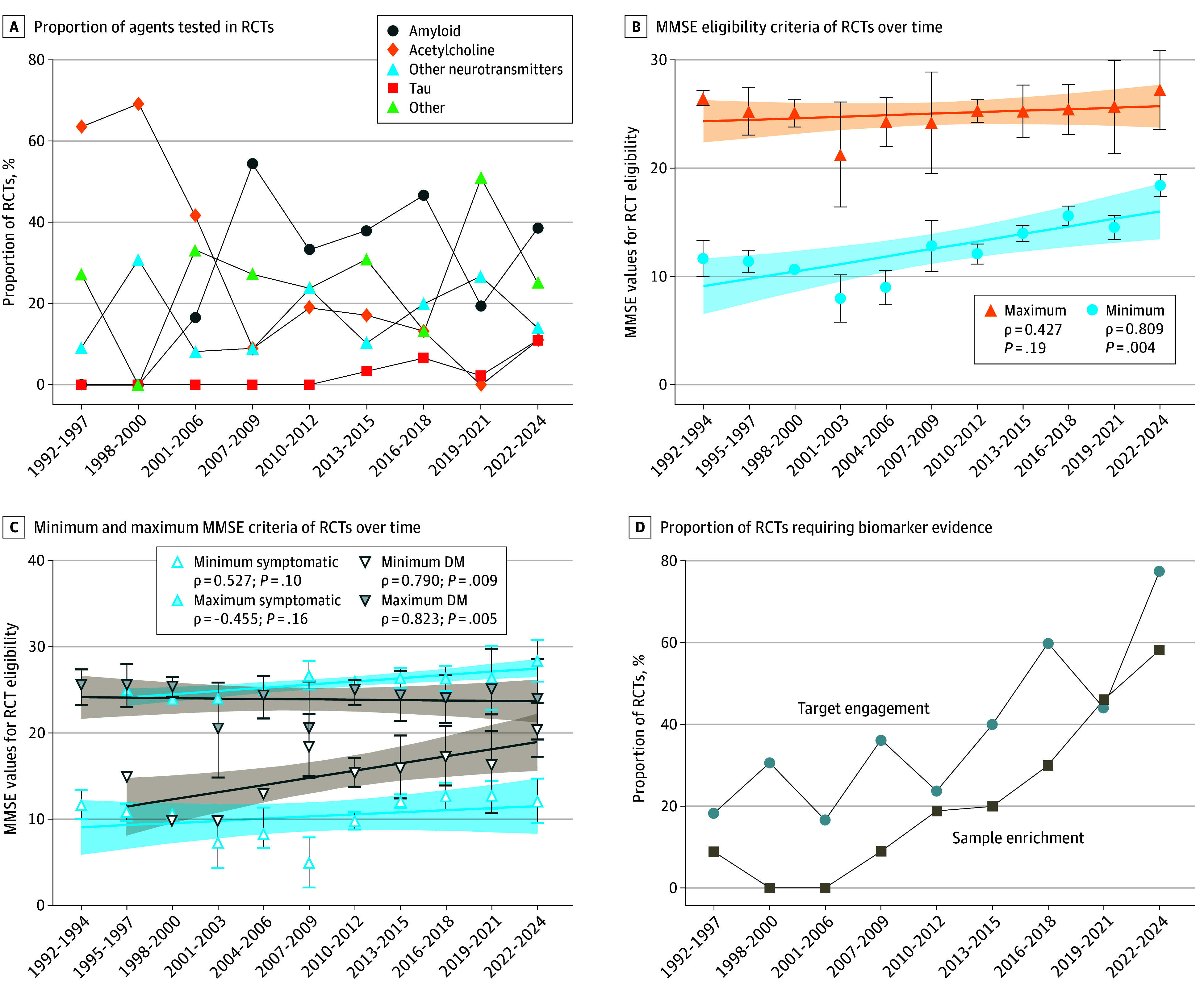
Trends in Randomized Clinical Trial (RCT) Targets and Measures Error bars show standard error to the mean, while shaded areas correspond to the 95% CIs of the regression lines. DM indicates disease modifying; MMSE, Mini-Mental State Examination.

The cholinergic system was the most common target in symptomatic trials, with 38 of 105 symptomatic RCTs (36.2%). Cholinergic system therapies accounted for 58.3% of 36 RCTs in the 1992 to 2006 period. In contrast, the 2007 to 2024 period was dominated by amyloid-targeting therapy, involving 35.9% of RCTs over that period (60 of 167 studies). However, this anti-amyloid dominance may be waning, as these trials represented only 19.5% of RCTs in the 2019 to 2021 period (8 of 41 studies), which is less than neurotransmitter-targeting trials (26.8% [11 of 41 studies]) for the first time since 2012 to 2015 (Figure 3A). We also noted the recent emergence of tau-targeting therapies (11.1% [4 of 36 studies] in 2022-2024), which might see future growth.

We next assessed the enrollment criteria used by RCTs. MMSE scores were the most frequent inclusion criteria, reported in 87.7% of RCTs (178 of 203 studies). Over time, the minimum and maximum MMSE scores have significantly increased (minimum: ρ = 0.818; *P* = .003; maximum: ρ = 0.655; *P* = .03). The range of mean MMSE scores included in trials from 1992 to 2006 was 9.88 to 24.48, and it was 14.93 to 25.72 from 2007 to 2024 (Figure 3B). More specifically, disease-modifying RCTs appeared to aim to recruit individuals with earlier-stage AD, while symptomatic RCTs did not significantly change over time (Figure 3C). In addition to cognitive screening tools, the use of AD biomarkers as inclusion criteria has increased from a marginal 2.8% in the 1992 to 2006 period (1 of 36 studies) to 35.7% in the 2007 to 2024 period (60 of 167 studies) (Figure 3D). In the 2007 to 2024 period, 50.0% of disease-modifying (47 of 94) and 17.8% of symptomatic (13 of 73) RCTs used AD biomarkers as inclusion criteria.

Mirroring the increase in biomarker use in inclusion criteria, RCTs increasingly used target engagement biomarkers, from 22.2% in the 1992 to 2006 period (8 of 36 studies) up to 49.7% in 2007 to 2024 (83 of 167) (Figure 3D). In the 2007 to 2024 period, 75.5% of disease-modifying (71 of 94) and 16.4% of symptomatic (12 of 73) RCTs used AD biomarkers for target engagement. The most common primary end points were cognition-related: 102 RCTs (50.2%) used the Alzheimer Disease Assessment Scale–Cognitive Subscale (ADAS-Cog), in addition to 44 studies (21.6%) using other cognitive outcomes. Disease-modifying RCTs tended to use the ADAS-Cog less frequently, and other cognitive tests more frequently than symptomatic RCTs in the 2007 to 2024 period (ADAS-Cog: 40.4% [38 of 94 studies] vs 61.6% [45 of 73 studies]; other cognitive end point: 36.1% [34 of 94] vs 20.5% [15 of 73]).

### RCT Reporting Practices

**Figure 4.**
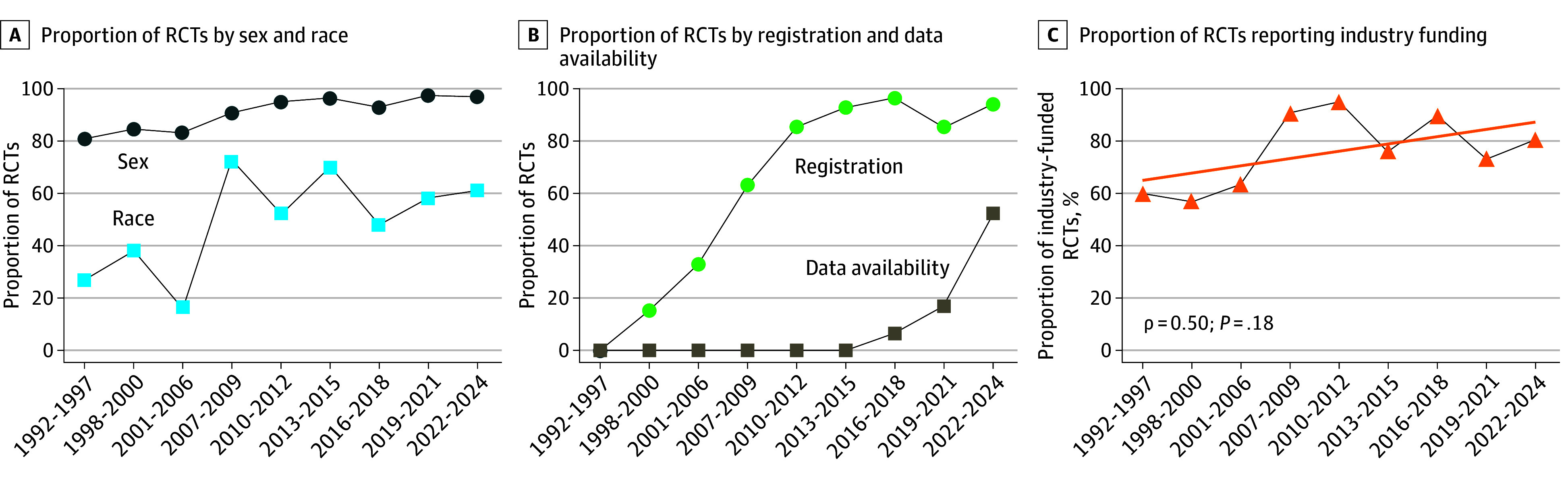
Trends in Randomized Clinical Trial (RCT) Reporting and Transparency Practices

Regarding reporting practices, 191 RCTs (93.6%) reported sex proportions, and 109 (53.4%) reported either the racial or ethnic composition of their sample. Of note, the racial and ethnic reporting practices have increased between the first and the second half of the study period (1992-2006: 27.8% [10 of 36]; 2007-2024: 59.5% [99 of 167]) (Figure 4A). Among studies reporting sex, 150 (78.5%) included 50% to 70% of women, 29 (15.2%) included 30% to 50% of women, and 12 (6.3%) included more than 70% of women. Among those reporting race, 104 (95.4%) had predominantly White samples, while 5 (4.6%) had predominantly East Asian samples. More than 80% of the sample was a part of the majority group in 104 studies (95.4%).

Open science practices showed similar trends to that of racial reporting. Registration to platforms such as ClinicalTrials.gov has drastically shifted from 16.7% (6 of 36 studies) in 1992 to 2006 to 89.2% (149 of 167 studies) in 2007 to 2024. The practice of sharing RCT data is more novel; it was first encountered in 2018, but it reached 51.4% (18 of 35 studies) between 2022 and 2024 (Figure 4B). However, data sharing conditions varied widely, ranging from being available upon request to granting open access to the anonymized dataset.

### RCT Funding Sources

We also evaluated the proportion of trials funded by the pharmaceutical industry vs not-for-profit groups over time, excluding studies not reporting study funding (last occurrence in 2001). We found an increase in the proportion of trials funded by the pharmaceutical industry with respect to time, but the difference was not statistically significant (ρ = 0.500, *P* = .18) (Figure 4C). The difference was mainly observed between the 1992 to 2006 period (60.0% [15 of 25 studies]) and the 2007 to 2024 period (82.1% [138 of 167 studies]), with no significant variation of the proportions within each period. In addition, specific therapeutic approaches were more likely to be funded by the pharmaceutical industry. For example, during the 2007 to 2024 period, anti-amyloid and anti-tau RCTs were predominantly industry-funded (95.8% [68 of 71 studies]) compared with more mixed funding sources in neurotransmitter-related therapies (77.6% [52 of 69]) and other therapeutic approaches (63.5% [33 of 52]) (χ^2^_2_ = 20.11; *P* < .001).

## Discussion

This study assessed the temporal trends in the design of AD RCTs from 1992 to the end of 2024. We found an increase in the sample size and duration of RCTs testing interventions in AD. We also found a shift toward the use of disease-modifying therapies over symptomatic therapies that might explain the increased study duration. We found an increase in the use of biomarkers to determine trial eligibility and assess target engagement as well as an increasing exclusion of patients with moderate to severe dementia defined by MMSE. Lastly, RCTs increasingly reported race and ethnicity, used open science practices, and received industry funding over time.

Some shifts in RCT design reported in our study are perceived as positive. Namely, the better reporting of race and ethnicity suggests greater efforts to recruit and retain diverse populations.^18^ Such reporting allows clinicians to make better inferences about the benefits their patients may receive from a specific treatment,^19^ but a greater consistency and standardization is needed. Further steps are necessary to truly represent global ethnocultural diversity, as most trials include an overwhelming majority of White participants.

The increased use of open science practices represents another positive change over the study period. AD RCTs followed a gradual increase in protocol registration during the 2000s, with most RCTs published after 2010 being registered. This pattern is also seen in RCTs from other fields of medicine due to journal and funder requirements.^20^ Clinical trial registration may help curtail *P *value hacking (ie, the misuse of statistical analyses to report significant findings) and questionable research practices, such as selective reporting.^21^ Therefore, the high prevalence of this practice increases the confidence in trial results for which the outcome and the analyses plan have been registered.

We additionally found a late emergence of data availability starting from 2018. This increase may reflect the International Committee of Medical Journal Editors (including *JAMA*, *Lancet*, and the *New England Journal of Medicine*) requirement to implement a data sharing statement since July 2018.^22^ Data sharing putatively improves transparency and may improve replicability and reproducibility. However, studies have shown that data sharing statements can be misleading. In one case, only 19% of the links from articles claiming that their data were available on a repository led to the dataset.^23^ Even worse, 93% of the authors of articles claiming that their data would be available upon reasonable request either did not respond or declined to share data.^24^ Therefore, the data availability statement should be tested before concluding that data sharing practices are growing.

We observed a marked increase in the sample size of AD RCTs. While greater sample sizes are undoubtedly positive to detect important safety signals and for statistical precision, the greater statistical power also allows for the detection of smaller clinical effects. Estimates for what constitutes clinically meaningful differences can vary according to stakeholder^25^ and according to disease severity.^26,27^ Because there is currently no consensus as to how to determine a clinically meaningful change in AD symptomatic progression, the drive toward detecting small effects may lead to therapies providing little therapeutic value. Our results therefore highlight the need to determine what constitutes a meaningful clinical difference for individuals living with AD and how this varies according to disease stage and severity.

We also found that trial durations have been increasing over time. Disease-modifying therapies would putatively have a cumulative effect over time which, over long durations, might more significantly improve the quality of life, as opposed to the rapid-onset but stable effects of symptomatic therapies.^28,29,30^ Therefore, disease-modifying therapies likely warrant longer trial durations. Our findings are consistent with this concept: we found that disease-modifying trials have seen a particularly robust duration increase, with recent phase 2 disease-modifying trials being longer than phase 3 symptomatic trials. This extended duration may therefore represent an adaptation of RCTs to the specific needs of disease-modifying trials. The increased durations mean that slowly occurring effects can be statistically significant. Therefore, the slow effect of disease-modifying therapies should be well communicated to clinicians and patients to avoid the perception of a lack of therapeutic effect.

The emergence of disease-modifying trials represents a shift from a clinical to a clinical-biological definition of AD starting around 2010.^31^ Rather than compensate for biological changes underlying symptoms, disease-modifying therapies generally target pathological mechanisms specific to AD. When testing such therapies, ascertaining whether individuals harbor AD pathology at the time of inclusion is believed to increase the likelihood of the trial’s success.^32,33,34^ This may explain the frequent use of biomarkers to determine eligibility in disease-modifying AD RCTs and to measure target engagement. Target engagement biomarkers may also offer the potential for partial success, reducing the risk of RCT failure. The aducanumab trials exemplify this: despite failing to achieve the primary end points in 1 of 2 phase 3 clinical trials, the significant amyloid-β clearance on positron emission tomography (PET) imaging was used to argue for approval by the US Food and Drug Administration.^35^ It is anticipated that the use of biomarkers to determine AD RCT eligibility will increase substantially due to advances in the accuracy of plasma biomarkers,^36,37,38^ which can either be used as a screening tool, as an inclusion criteria, or eventually as surrogate primary end points.^34^

Since 2007, the proportion of industry-funded trials has been greater than 80%. This hints that public funding for RCTs has not followed their rising costs and may have become insufficient.^39^ Indeed, the sample size and duration of RCTs as well as the use of costly screening tools (eg, PET imaging) have also grown over this same period, drastically increasing the average cost of an RCT. This is supported by our findings, where industry-funded RCTs increased in sample size and duration while not-for-profit-funded RCTs did not.

### Limitations

This study has limitations. The main limitation is that we only assessed peer-reviewed published RCTs, which could result in a publication bias. Specifically, positive RCT results are more likely to be published than negative ones.^40^ Although we did not directly examine the outcomes per se, RCTs with weaker statistical power may be at increased risk of remaining unpublished. Nevertheless, in contrast to other studies that focused on RCTs from specific high-impact (so-called practice-changing) journals, our study did not limit the search to specific sources. The publication bias of the current study may therefore be smaller in comparison with studies in other areas of medicine.^6,9^ Our exclusion of most nonpharmaceutical RCTs due to their unblinded design limits generalizability, as these trials may follow different design trends and attract less industry funding. In addition, since the outcomes themselves were not assessed, publication bias has a much smaller impact on our results and interpretation than for meta-analyses. Our study also classified trials as disease modifying based on their purported therapeutic mechanisms. However, the complete determination of therapeutic mechanisms for investigated compounds cannot be fully known.^41^

## Conclusions

In this study of AD RCTs, we show changes in RCTs’ design and key features over the past three decades in response to an increasingly biological definition for AD and the emergence of new AD therapeutic approaches. There has been a significant shift from symptomatic to disease-modifying trials, exemplified by cholinergic system therapies and amyloid-β treatments, respectively. This transition was associated with increased duration of RCTs over time, which in turn has likely contributed to a sharp increase in RCTs costs, amplifying the need for industry funding. The use of target-engagement and sample-enrichment biomarkers to characterize the biological changes associated with AD could reduce the risks of trial failure. Clinicians and patients will need to be informed of the subtler and slowly occurring effects of disease-modifying therapies, and more work will be needed to fully understand their full value.

## Notes

Supplement 1. **eFigure.** Number and Mechanism of Action of RCTs Included in the Study Across the Time Window

Supplement 2. **eTable.** Included RCTs

Supplement 3.
Data Sharing Statement
