# Supplementary material for: The Evolution of Randomized Clinical Trial Designs to Assess Therapeutics in Alzheimer Disease
Source: JAMA Netw Open. 2025 Aug 29;8(8):e2529665. doi: 10.1001/jamanetworkopen.2025.29665 (PMC12397894; doi:10.1001/jamanetworkopen.2025.29665)
Supplement: Supplement 1. — eFigure. Number and Mechanism of Action of RCTs Included in the Study Across the Time Window [file jamanetwopen-e2529665-s001.pdf]

Supplemental Online Content

Aumont E, Therriault J, Kwan AH, et al. The evolution of randomized clinical trial designs to assess therapeutics in Alzheimer disease. *JAMA Netw Open.* 2025;8(8):e2529665. doi:10.1001/jamanetworkopen.2025.29665

eFigure. Number and Mechanism of Action of RCTs Included in the Study Across the Time Window

This supplemental material has been provided by the authors to give readers additional information about their work.

© 2025 Aumont E et al. *JAMA Network Open*.

eFigure. Number and Mechanism of Action of RCTs Included in the Study Across the Time Window

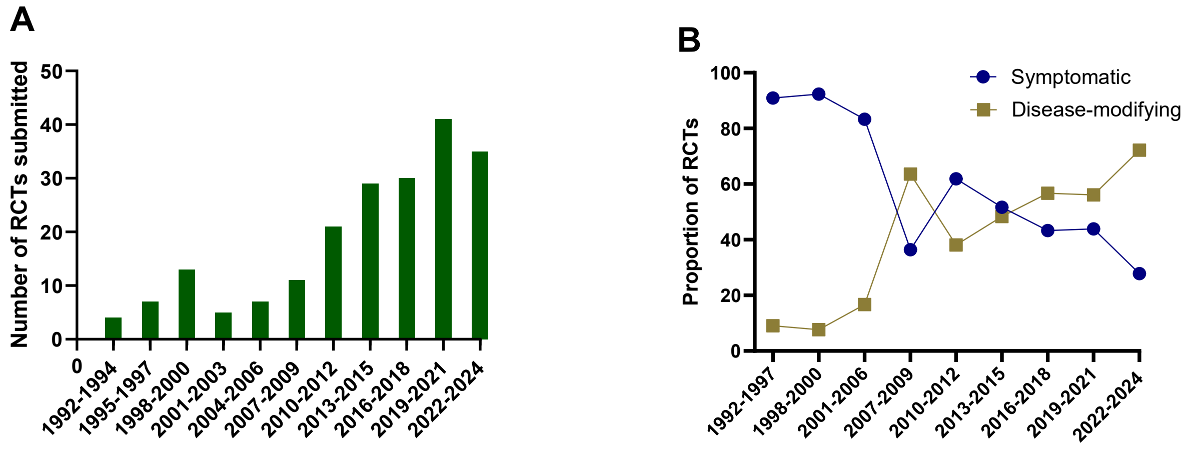

A: The number of studies published robustly increased over time. 1992-1994 and 1995-1997 periods as well as 2001-2003 and 2004-2006 periods were grouped by pairs in frequency analyses due to the low study count. **B:** Proportion of AD RCTs described as either symptomatic or disease-modifying. Disease-modifying RCTs became more prevalent starting from 2007. RCTs: Randomized clinical trials.

© 2025 Aumont E et al. *JAMA Network Open*.
