# Supplementary material for: The Evolution of Randomized Clinical Trial Designs to Assess Therapeutics in Alzheimer Disease
Source: JAMA Netw Open. 2025 Aug 29;8(8):e2529665. doi: 10.1001/jamanetworkopen.2025.29665 (PMC12397894; doi:10.1001/jamanetworkopen.2025.29665)
Supplement: Supplement 3. — Data Sharing Statement [file jamanetwopen-e2529665-s003.pdf]

Data Sharing Statement

Aumont. The Evolution of Randomized Clinical Trial Designs to Assess Therapeutics in Alzheimer Disease. *JAMA Netw Open*. Published August 29, 2025.
doi:10.1001/jamanetworkopen.2025.29665

Data
Data available: Yes
Data types: Data (not involving human participants), Data dictionary
How to access data: The full dataset is available on OSF (https://osf.io/5ghaq/files/osfstorage/6835d7349e9931ebe8a4db44).
When available: With publication

Supporting Documents
Document types: None

Additional Information
Who can access the data: Anyone
Types of analyses: For any purpose
Mechanisms of data availability: Downloading the data on OSF
